# Activity-based protein profiling: A graphical review

**DOI:** 10.1016/j.crphar.2023.100164

**Published:** 2023-08-24

**Authors:** Exequiel O.J. Porta, Patrick G. Steel

**Affiliations:** Department of Chemistry, Durham University, Durham, DH1 3LE, United Kingdom

**Keywords:** Activity-based protein profiling, Activity-based probes, Affinity-based probes, Chemical probes, Chemoproteomics, Drug discovery, Mass spectrometry, Target discovery

## Abstract

Activity-based protein profiling (ABPP) is a chemoproteomic technology that employs small chemical probes to directly interrogate protein function within complex proteomes. Since its initial application almost 25 years ago, ABPP has proven to be a powerful and versatile tool for addressing numerous challenges in drug discovery, including the development of highly selective small-molecule inhibitors, the discovery of new therapeutic targets, and the illumination of target proteins in tissues and organisms. This graphical review provides an overview of the rapid evolution of ABPP strategies, highlighting the versatility of the approach with selected examples of its successful application.

## 1 Introduction

Activity-based Protein Profiling (ABPP) is a highly flexible and powerful chemoproteomic technology that utilizes small molecule probes, known as activity-based probes (ABPs), to react with the active sites of proteins selectively and covalently. The labelled proteins can then be captured and analyzed using a variety of proteomic tools. This technique enables the analysis of protein functional states in complex biological systems, including intact cells and animal models, in a global and quantitative manner (Speers and Cravatt, 2009). Arguably, the origins of the technique arose in the covalent affinity chromatography experiments used in the 1970's to isolate penicillin-binding proteins (Blumberg and Strominger, 1974). However, the modern concept of an ABPP experiment was first described in the late 1990s (Bogyo et al., 1997; Liu et al., 1999). Since then, it has become globally and widely used by the scientific community. Originally employed as a largely qualitative technique, rapid advances in mass spectrometry platforms have not only significantly increased sensitivity but also enabled quantitative analyses.

Because ABPs selectively label active enzymes, rather than their inactive forms, this facilitates the characterization of changes in enzyme activity that take place without alterations in protein levels. This makes ABPP a valuable tool that complements conventional genetic experiments and other ‘omic’ methods for biological discovery (Galmozzi et al., 2014).

ABPP has had an extraordinary impact in the field of drug discovery and our understanding of fundamental biology concepts (Cravatt et al., 2008). Through its ability to selectively profile proteins within complex proteomes, ABPP has been instrumental in identifying new therapeutic targets and developing highly selective small-molecule inhibitors. Additionally, ABPP has facilitated the discovery of previously uncharacterized enzymes, expanding our understanding of enzymatic activities in cells and organisms. In this graphical review, we will discuss the key components, workflows, main uses, functionalities, and future directions of this powerful technology.

## 2 In all ABPP workflows, the correct probe design is the cornerstone

**Fig. 1:**
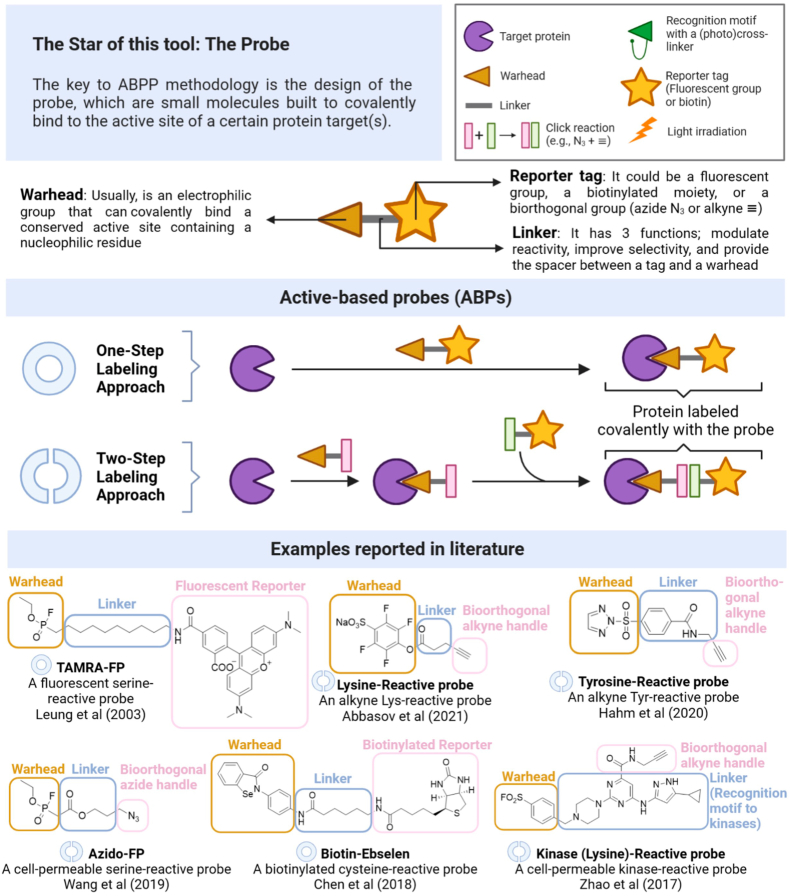
(**Top**) The central component of ABPP is the probe. Commonly, an ABP is comprised by three parts: a warhead, a linker, and a reporter. ABPs are equipped with an electrophilic reactive moiety (warhead) that is specifically tailored to covalently label the catalytically active nucleophilic residues of particular proteins or protein families in a selective and irreversible manner. (**Medium**) There are two methods available for labelling: one-step labelling and two-step labelling. In the one-step labelling approach, the probe already contains the reporter group, whilst in the two-step labelling method, the reporter unit is added in a post-labelling step. This method is especially advantageous when direct ABPP using fluorescent or biotinylated probes proves ineffective (e.g., due to cell permeability). (**Bottom**) Selected examples of probes that feature distinctive components (Abbasov et al., 2021; Chen et al., 2018; Hahm et al., 2020; Leung et al., 2003; Wang et al., 2019; Zhao et al., 2017).

**Fig. 2:**
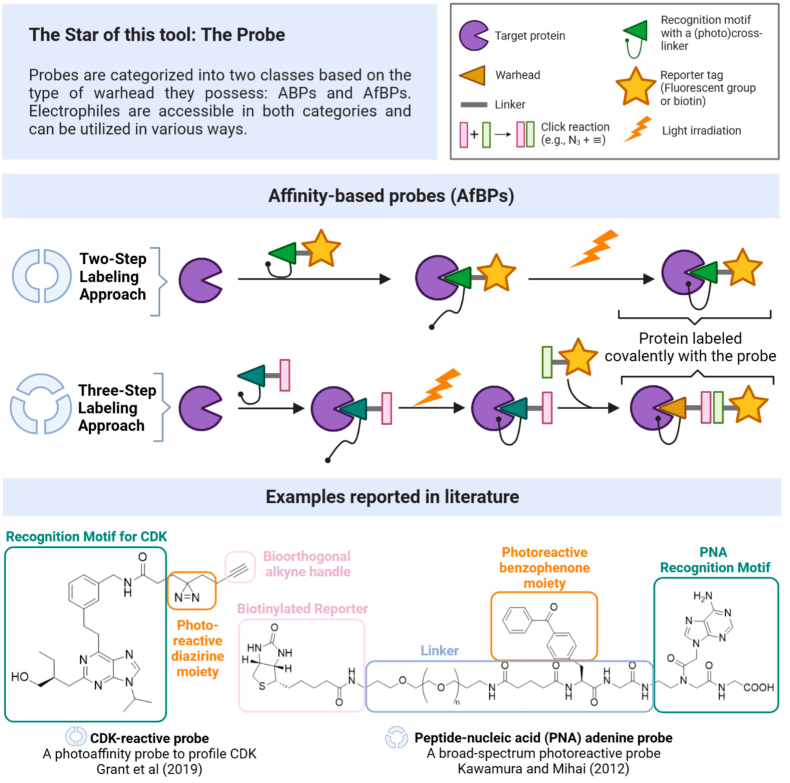
An affinity-based probe or AfBP contains a highly selective recognition motif coupled with a photo-affinity motif, typically a benzophenone or a diazirine group, that covalently binds to the target protein following irradiation (generation of an electrophilic radical). In this case, unlike ABP, AfBP requires an additional step, which involves irradiation. Consequently, there are two potential strategies for labelling: a two-step approach involving incubation followed by irradiation, or a three-step method comprising incubation, irradiation, and subsequent bioorthogonal chemistry. In the **bottom**, two reported examples that feature distinctive AfBP components (CDK is cyclin-dependent kinases, PNA is Peptide Nucleic Acid) (Grant et al., 2019; Kawamura & Mihai, 2012).

The key to the ABPP methodology lies in the design of the probe, which consists of small molecules specifically engineered to covalently bind to the active site(s) of certain protein(s). Probes can be divided into two classes depending on the nature of the warhead: Activity-based chemical probes (ABPs – Fig. 1) and affinity-based chemical probes (AfBPs – Fig. 2) (Fang et al., 2021). ABPs contain an electrophilic reactive group designed to irreversibly and selectively label the catalytically active nucleophilic residues of specific proteins or protein families. Alternatively, AfBPs contain a highly selective recognition motif coupled with a photo-affinity group that labels its cognate target protein upon UV-irradiation. The main distinction between ABPs and AfBPs lies in their selectivity. ABPs react specifically with related classes of enzymes relying on a common mechanism of action, such as the catalytic triad of the serine hydrolases; whereas AfBPs, upon activation, interact with any nearby nucleophilic residue with selectivity for the specific target protein achieved through a classical ligand-protein binding interaction. Consequently, prior knowledge of the target is required for AfBPs, whilst mechanistic knowledge is needed for design of ABPs.

**Fig. 3:**
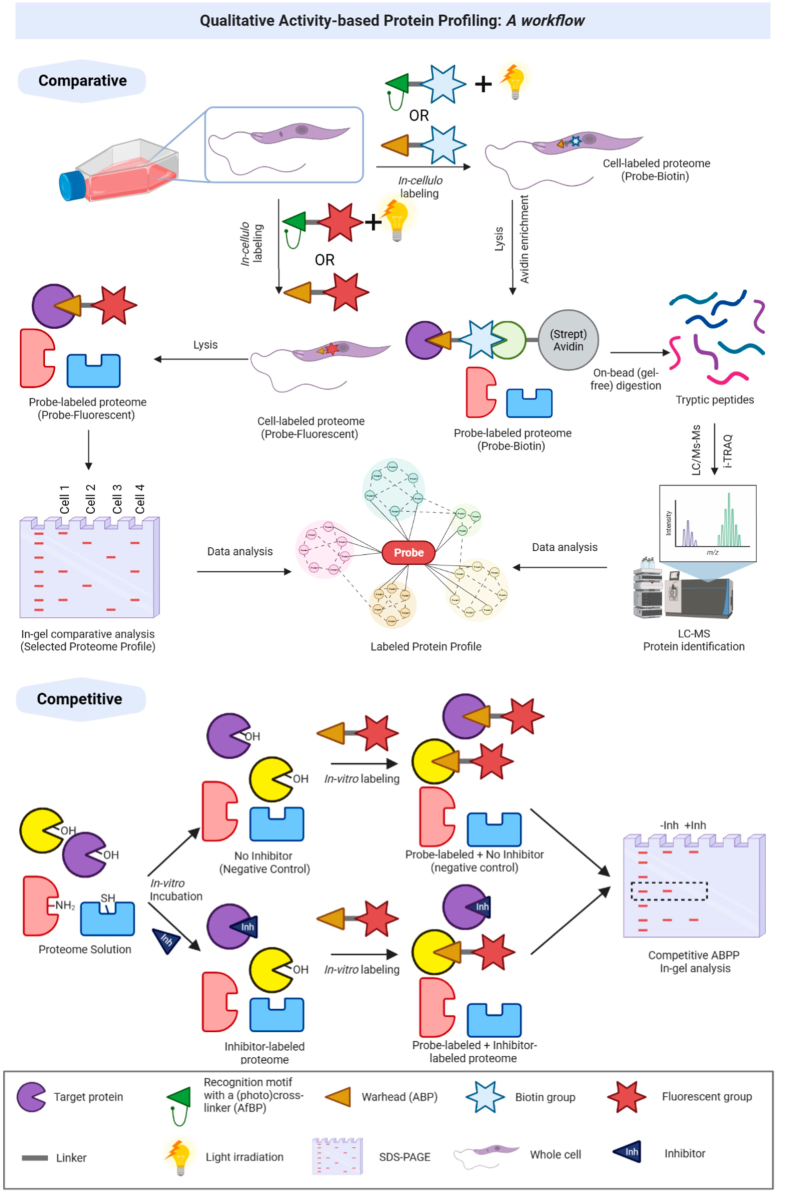
By employing A(f)BPs, qualitative ABPP allows for functional annotations of proteins within an organism to be acquired. This approach is often the fastest and most cost-effective method for profiling a proteome. (**Top**): A model workflow for qualitative ABPP, including comparative ABPP (e.g., comparing the targeted proteome profile of different cell lines) for identifying biomarkers and targets. In-gel experiments (left), LC-MS approaches (right), or their combinations can be employed. (**Bottom**): Competitive ABPP experiments for identifying inhibitors. In this case, the example uses probes targeting active-site serine residues, such as those found in the serine hydrolases. Briefly, the proteome is first preincubated with the inhibitor and then incubated with the probe. If the inhibitor successfully blocks the probe's binding site, a decrease (or complete disappearance) in the fluorescence intensity for that protein will be observed.

**Fig. 4:**
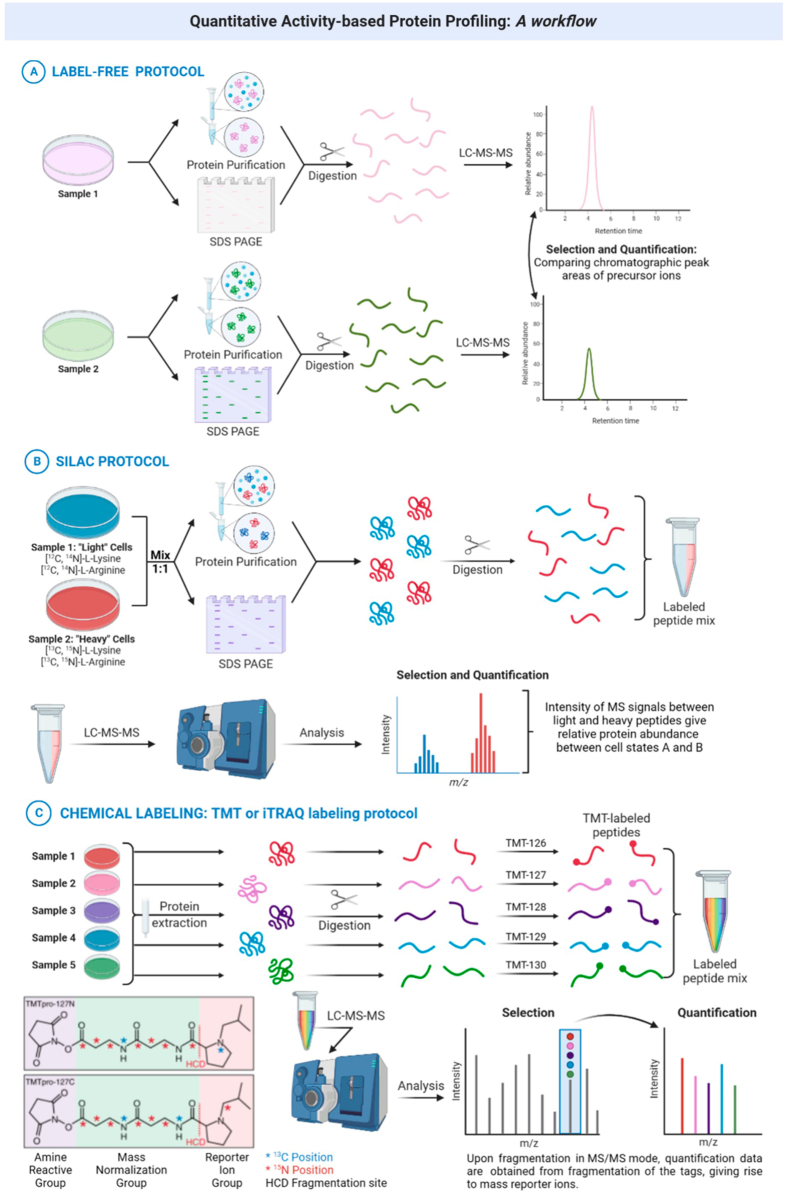
Utilizing cutting-edge quantitative chemoproteomics tools, quantitative ABPP-MS workflows offer a powerful approach to quantify proteins labelled with ABPs. This enables researchers to measure changes in functional activity at specific protein sites, leading to valuable insights into protein regulation and functionality, and providing a comprehensive understanding of the dynamics and alterations in protein activities. These innovative methods allow for the precise measurement of ABP-labelled proteins, revealing how they respond to various stimuli and molecular interactions. Different techniques for applying quantitative ABPP are shown, including label-free (**A**), SILAC (**B**), and chemical labeling (**C**) experiments. Each method has its advantages and disadvantages (cost, time, complexity, multiplexability, etc.), and has to be selected according to the experimental model that needs to be studied (Stepath et al., 2020).

**Fig. 5:**
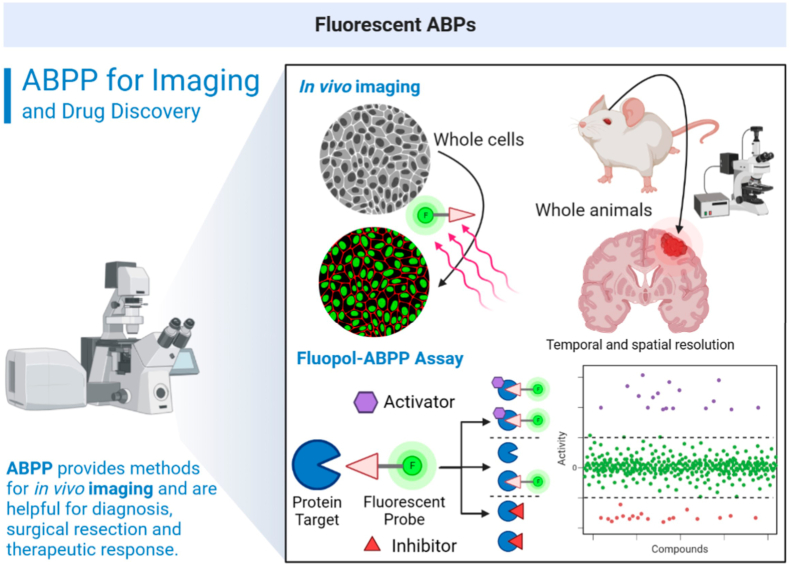
Fluorescent probes can be used as important tools for the diagnosis and treatment of various diseases. To achieve this, these probes must possess desirable pharmacokinetic, pharmacodynamic, and physicochemical properties, enabling them to penetrate biological barriers and reach their targets. This capability allows them to use for illuminating molecular targets in both cells and whole organisms with spatial and temporal resolution (**top**). Another important use of these probes is in the search for inhibitors or activators of certain molecular targets. One technique, known as FluoPol-ABPP, can be employed for high-throughput screening (**bottom**) and is an ideal approach for studying enzymes for which insights into structure and substrates are not yet known. (FluoPol – fluorescence polarization).

The essential structure of an ABP commonly includes three parts: 1) a reactive group or warhead, usually an electrophilic group that can covalently bind an active site containing a nucleophilic residue of mechanistically related classes of enzymes; 2) a linker region, which has three functions, namely, modulating the reactivity of the warhead, enhancing selectivity to the binding of the probe to the target site, and providing a spacer between the warhead and the reporter tag; and 3) a reporter tag, providing a handle to detect, manipulate and/or measure labelled protein, by microscopy, SDS-PAGE, or mass spectrometry analysis. The reporter tags typically consist of a fluorophore, an affinity tag, or sometimes a combination of both. However, for *in cellulo* applications, these tags can hinder probe uptake, leading to the preference of small bioorthogonal functional groups like alkynes or azides that enable post-labeling conjugation with a tagging agent. Consequently, two labelling options are available: a one-step labelling strategy and a two-step process. For the two-step process, bioorthogonal click chemistry utilizes these small functionalities, which can react with complementary azide or alkyne partners (or other pairs of chemical reporters), enabling the attachment of a fluorophore or an affinity agent. These reactions significantly improve probe cell permeability by replacing bulky groups (e.g., fluorescent reporters or biotin) with smaller alkyne or azide groups and allow easy diversification of a probe with various reporter groups through a single bioorthogonal reaction. While several bioorthogonal reactions are available (Verhelst et al., 2020), the copper (I)-catalyzed azide-alkyne cycloaddition (CuAAC) click chemistry reaction stands out as the most widely used, (Kolb et al., 2001). For *in cellulo* analysis, strained alkynes can enable copper-free cycloaddition reactions, eliminating the cytotoxicity associated with copper (Bertozzi, 2009).

## 3 The essential component of ABPP workflows

The ABPP workflow begins with the design and synthesis of the probe. Once the probe is synthesized, it is incubated with the analyte of interest, which can be a cell fraction, whole cells, tissues, or animals, to covalently react with the target protein(s). An important initial step in this process is to identify the optimal conditions for the specific assay, considering factors such as the nature of the analyte (e.g., whole-cell or lysate incubation, along with the lysis condition for the latter), probe toxicity, concentration, and incubation time. The selection of a reporter system is also critical, as discussed in the following sections. With these parameters established, assays are undertaken, and the outputs are visualized using qualitative and quantitative detection platforms. These platforms include polyacrylamide gel electrophoresis (SDS-PAGE) followed by fluorescence scanning or Western blotting to identify the target(s), liquid chromatography-mass spectrometry (LC-MS), or imaging using fluorescent microscopy. Each method has its own benefits and limitations, and as a result, they are often used in combination (Wang et al., 2018). For instance, gel-based detection methods are suitable for high throughput analyses, allowing rapid comparative and competitive analysis of multiple proteins simultaneously. However, they have limitations in terms of resolvability and accuracy, as gel bands may contain multiple proteins. To address this challenge, various LC-MS strategies have been introduced, significantly improving the precision of target identification. A combination of gel-based and LC-MS methods is considered ideal for target identification. Fluorescent ABPs are used for rapid screening via in-gel analysis, while streptavidin enrichment, pull-down, and mass spectrometry analysis using biotin ABPs are employed for target identification.

Once successful identification of protein target(s) by ABPP approaches has been achieved, it is necessary to validate this result. A variety of strategies can be employed; namely, the use of recombinant proteins to (re)execute the ABPP workflow and validate the results (feedback); System interrogation with known inhibitors in competition with the probe (competitive ABPP); Genetic validation by CRISPR-Cas9, mutagenesis, gene deletion, etc., complementarily followed by ABPP; biophysical strategies; among others.

## 4 Workflows identifying targets labelled by A(f)BPs: Qualitative approaches (Fig. 3)

In qualitative ABPP, the probe labelled proteins are coupled with a tag that enables isolation (e.g., biotin-streptavidin) and/or visualization (e.g., a fluorophore). As such, qualitative ABPP enables the acquisition of functional annotations of proteins within an organism. This approach is typically the fastest and most cost-effective method for profiling a proteome. When used in a competitive ABPP workflow target identification becomes possible.

In qualitative ABPP, the simplest and most common method for target protein visualization is to utilize gel electrophoresis to separate proteins by one-dimensional (1D)– or two-dimensional (2D)–PAGE and detect the proteins by in-gel fluorescence scanning (fluorescent reporters). Other less common forms of visualization include streptavidin blotting, Western blotting, and immunoprecipitation. With the exception of Western blotting, this only gives an indication of the size of the protein(s) labelled by the probe. Target identification using in-gel fluorescence analysis most commonly employs comparative or competitive ABPP techniques, as these represent simple, efficient and cheap methodologies to discover and validate targets (Deng et al., 2020). Comparative analysis involves the correlation and profile of the output with (a set of) known proteins run on the same gel, whilst competitive assays employ selective small molecules that compete with the probe for the same active site, thus providing a strong association to a specific protein. In addition to target identification, these techniques also have many other useful outputs. For instance, comparative studies of two distinct biological samples, such as healthy and disease samples, can reveal variations in enzyme activity, providing powerful platforms to identify biomarkers, whereas competitive assays can be a powerful tool for screening potential inhibitors against a new identified target. However, while these qualitative methods are robust and suitable for rapid profiling, there are limits in sensitivity, resolution, and accuracy. For example, a single band in a gel may contain multiple proteins.

The limitations in gel-based methods can be addressed by LC-MS-based methods, owing to their high sensitivity and resolution, especially for the identification of low-expressed proteins, and have emerged as the standard approach for ABPP strategies. The simplest (and original) approach involves the excision of labelled bands from SDS-PAGE gels, followed by digestion with trypsin, and then analysis of the tryptic peptides by LC-MS, with identification achieved by comparison with protein sequence databases. However, this still exhibits the sensitivity limitations of a gel-based protocol, and gel-free processes are considered to be more efficient. In gel-free approaches, the treatment of a complex proteome with a biotinylated ABP is followed by enrichment of the probe-labelled proteins by incubation with (strept)avidin beads, on-bead digestion, and analysis by LC-MS as above.

## 5 Workflows using quantitative chemoproteomics to identify the functional state of a proteome: Quantitative approaches (Fig. 4)

Ultimately, the functionality of a protein is determined by a combination of activity and expression. When combined with modern quantitative chemoproteomics tools, ABPP-MS workflows can be employed to quantify ABPs-labelled proteins and measure changes in functional activity, providing valuable functional insights into protein regulation. Furthermore, competitive quantitative ABPP can be used to determine the global selectivity profiles of tool compounds and drugs in lysates, cells, and animals. The coupling with quantitative chemical proteomics enables high-throughput ABPP methods which can also improve the accuracy of target-protein identification. A number of quantitative chemical proteomic approaches have been applied in this way (Wong et al., 2017), including metabolic labeling (SILAC), chemical labeling (iTRAQ and TMT), and label-free approaches (Chen et al., 2017).

The simplest approach and one that requires minimal interventions is to use label-free quantitative proteomics measuring protein abundance using mass spectrometric signal intensities. This approach is cost-efficient and widely applicable but has limitations in terms of dynamic range and precision of quantification, particularly with highly promiscuous compounds (probes) and samples containing highly abundant proteins. Label-free methods also require separate MS runs for each sample, which introduces additional variance.

Stable Isotope Labeling by Amino acids in Cell culture (SILAC) addresses the issue of low sensitivity and provides more reliable reproducible results. This involves culturing two different cell populations, one in a medium containing “light” amino acids and the other in a medium containing “heavy” (isotopically labelled) amino acids (Chen et al., 2015). The “heavy” amino acids are incorporated into newly synthesized proteins, enabling relative comparisons to be made between “heavy” and “light” samples by comparing the ratio of ion intensities of each sample. However, the method requires the system under analysis to be amenable to culture and metabolic labeling and is limited to comparative studies of very few samples at a time.

Far greater throughput (multiple samples in a single run) can be achieved using chemical labeling approaches. These also enable the quantification of proteins in systems that cannot be metabolically labelled, such as natural microbial communities or primary tissue samples. A number of protocols have been described, with the current state-of-the-art being the isobaric labeling methods, such as the Isobaric Tag for Relative and Absolute Quantification (iTRAQ) and Tandem Mass Tag (TMT) methods (Rauniyar and Yates, 2014). In iTRAQ or TMT experiments, following enrichment and digestion as before, the tryptic peptides are labelled with different iTRAQ or TMT reagents, which have identical masses but different fragmentation patterns. The samples are pooled, and a single MS analysis now provides both protein identity and relative quantification between different samples.

## 6 Fluorescent ABPs for cellular imaging and high throughput screening (Fig. 5)

Imaging agents that facilitate *in vivo* visualization and quantification have immense potential for monitoring chemotherapy outcomes, as well as for early diagnosis and disease monitoring. For those, fluorescent ABPs can be utilized to visualize and localize enzyme activity, within cells or whole organisms, by fluorescent microscopy. These require cell-permeable fluorescent probes or in-cell click fluorescent reporters. A critical and often limiting requirement is that all components have pharmacokinetic and pharmacodynamics properties suitable for *in vivo* studies.

For whole organism imaging, several techniques such as one and two photon near-IR imaging (NIRF) and positron emission tomography (PET) can be used. By combination of fluorescent probes with non-overlapping emission spectra, it is possible to simultaneously analyze multiple proteins in a multiplexed manner. PET-ABPs and NIRF-ABPs are potentially valuable *in vivo* imaging agents for disease diagnosis, for the identification of specific therapeutic targets and biomarkers, and for monitoring the efficacy of small-molecule inhibitors (Lee and Bogyo, 2010; Hight et al., 2014). These technologies will find growing applications in the future.

Fluorescent probes also have a significant potential for high-throughput screening (HTS) of targets with poorly characterized substrate or biological functions, for which traditional screening methods are not applicable. For instance, fluorescent polarization FluoPol-ABPP HTS assay (Knuckley et al., 2010) combines fluorescent probes with competitive inhibition strategies to identify inhibitors of enzymes that may not yet have been fully characterized. The screening assay relies on monitoring changes in fluorescence polarization emission of a free probe compared to a protein bound probe. The former, being smaller, rotates at a faster rate producing depolarized light and a lower (or no) FluoPol signal. In a competitive ABPP experiment, the addition of an inhibitor of a FluoPol-ABP will therefore lead to less bound probe and a concomitant reduction in signal. As signal is dependent on the amount of bound probe, this eliminates the need to wash away excess probes, enabling significantly higher throughput and the screening of lower affinity interactions. Therefore, FluoPol-ABPP is an efficient method for identifying enzyme inhibitors without prior knowledge of structure, substrate, or even biological function.

## 7 Summary and future perspectives

ABPP is a highly flexible and powerful chemoproteomic technology that enables the identification of proteins that can be pharmacologically interrogated. ABPP combines ABPs and qualitative and quantitative proteomics tools to help to understand the mode of action of compounds and proteins in complex (ideally native) proteomes. Furthermore, the use of isobaric reagents such as TMT or iTRAQ has increased the sensitivity of target quantification, making it easier to identify low-expressed protein targets in a multiplexed way. As such, ABPP underpins a range of applications, from target identification to drug discovery (Benns et al., 2021) and *in situ* and *in vivo* bio-imaging (Xu et al., 2020). Competitive ABPP and comparative ABPP have significantly impacted many stages of drug discovery. Comparative ABPP is particularly useful for target identification and validation (Morimoto and van der Hoorn, 2016), while competitive ABPP is a powerful tool for discovering new inhibitors for specific targets (Porta et al., 2022). By enabling target visualization in living cells or animals, fluorescent probes have enormous potential for future disease diagnosis and therapeutics.

Over the past 25 years, the wide use of ABPP in medicinal chemistry and chemical biology has been firmly established (Porta, 2023). However, there is still room for growth and innovation in the field. The generation of new detection methods to ABPP will broaden the applications of this methodology to other areas. Additionally, combining ABPP with other chemical proteomic technologies (and with ‘multiomic’ approaches) will expand the universe of the druggable proteome. Developing new and selective probes for unexplored protein families will be an essential objective for *in vitro* and *in vivo* measurement of protein activity in physiological and disease settings and will significantly accelerate drug development processes. Notably, despite the significant strides made in the last two decades, there are still a vast number of druggable targets that remain unexplored and lack a suitable chemical probe. Only a meager proportion (less than 20%) of the human proteome has a well-defined structure, let alone a ligand or a discernible ligand-binding site. Many of these proteins will not have a catalytic function and thus the biggest advances are likely to come through the expansion of the A(f)BPs pool. These developments are likely to be matched by further advances in the accompanying analytical methods, so the future is positive (Spradlin et al., 2021).

In conclusion, ABPP strategies have provided valuable platforms in medicinal chemistry and chemical biology. As we move forward, ABPP will continue to facilitate drug discovery processes, from target validation to drug candidate development, whilst also providing the scientific community with key insights into fundamental biological processes, ensuring its ongoing relevance in the field.

## CRediT authorship contribution statement

**Exequiel O.J. Porta:** Conceptualization, Methodology, Investigation, Writing – original draft, Writing – review & editing, Visualization. **Patrick G. Steel:** Conceptualization, Writing – original draft, Writing – review & editing, Funding acquisition, Supervision.

## Declaration of competing interest

The authors declare that they have no known competing financial interests or personal relationships, which have, or could be perceived to have, influenced the work reported in this article.

## Data Availability

No data was used for the research described in the article.
